# A Computational Framework for Proteome-Wide Pursuit and Prediction of Metalloproteins using ICP-MS and MS/MS Data

**DOI:** 10.1186/1471-2105-12-64

**Published:** 2011-02-28

**Authors:** W Andrew Lancaster, Jeremy L Praissman, Farris L Poole, Aleksandar Cvetkovic, Angeli Lal Menon, Joseph W Scott, Francis E Jenney, Michael P Thorgersen, Ewa Kalisiak, Junefredo V Apon, Sunia A Trauger, Gary Siuzdak, John A Tainer, Michael W W Adams

**Affiliations:** 1 Department of Biochemistry and Molecular Biology, University of Georgia, Athens, GA 30602, USA; 2 Philadelphia College of Osteopathic Medicine, Suwanee, GA 30024, USA; 3 Scripps Center for Mass Spectrometry and the Departments of Molecular Biology and Chemistry, The Scripps Research Institute, La Jolla, CA, 92037, USA; 4 Life Sciences Division, Lawrence Berkeley National Laboratory, Berkeley, CA 94720, USA

## Abstract

**Background:**

Metal-containing proteins comprise a diverse and sizable category within the proteomes of organisms, ranging from proteins that use metals to catalyze reactions to proteins in which metals play key structural roles. Unfortunately, reliably predicting that a protein will contain a specific metal from its amino acid sequence is not currently possible. We recently developed a generally-applicable experimental technique for finding metalloproteins on a genome-wide scale. Applying this metal-directed protein purification approach (ICP-MS and MS/MS based) to the prototypical microbe *Pyrococcus furiosus *conclusively demonstrated the extent and diversity of the uncharacterized portion of microbial metalloproteomes since a majority of the observed metal peaks could not be assigned to known or predicted metalloproteins. However, even using this technique, it is not technically feasible to purify to homogeneity all metalloproteins in an organism. In order to address these limitations and complement the metal-directed protein purification, we developed a computational infrastructure and statistical methodology to aid in the pursuit and identification of novel metalloproteins.

**Results:**

We demonstrate that our methodology enables predictions of metal-protein interactions using an experimental data set derived from a chromatography fractionation experiment in which 870 proteins and 10 metals were measured over 2,589 fractions. For each of the 10 metals, cobalt, iron, manganese, molybdenum, nickel, lead, tungsten, uranium, vanadium, and zinc, clusters of proteins frequently occurring in metal peaks (of a specific metal) within the fractionation space were defined. This resulted in predictions that there are from 5 undiscovered vanadium- to 13 undiscovered cobalt-containing proteins in *Pyrococcus furiosus*. Molybdenum and nickel were chosen for additional assessment producing lists of genes predicted to encode metalloproteins or metalloprotein subunits, 22 for nickel including seven from known nickel-proteins, and 20 for molybdenum including two from known molybdo-proteins. The uncharacterized proteins are prime candidates for metal-based purification or recombinant approaches to validate these predictions.

**Conclusions:**

We conclude that the largely uncharacterized extent of native metalloproteomes can be revealed through analysis of the co-occurrence of metals and proteins across a fractionation space. This can significantly impact our understanding of metallobiochemistry, disease mechanisms, and metal toxicity, with implications for bioremediation, medicine and other fields.

## Background

Metallomics is an emerging field that seeks to comprehensively characterize the role of metals in organisms [1]. As with any new field, unique challenges have been encountered in terms of experimental methodologies and data analysis. The essential role metals play in biology has long been appreciated, but the complete metallome of any organism has yet to be characterized. It is estimated that around a third of all proteins in an organism require a metal partner [2]. While it is possible to predict that certain proteins contain a metal [3], there are fundamental limitations to all current computational methods in comprehensively describing the metalloproteome of any organism. Many current methods rely on sequence motifs that in turn depend on the existence of a sufficiently sized set of previously annotated homologous proteins, a problem further compounded by the diversity of metal-binding sites across organisms as well as within a single organism [4]. It is also well known that heterologous protein expression can result in the production of proteins incorporating metals that are not natively incorporated [5]. In addition, while certain proteins and protein families are known to bind a variety of metals and are annotated accordingly, many are annotated as binding a single metal based on limited evidence.

It was shown recently [6] that the set of metals known to interact with proteins in vivo is a significant underestimate of the true extent and diversity of the metalloproteome. The approach developed used metal-directed protein purification relying on inductively coupled plasma mass spectrometry (ICP-MS) and tandem mass spectrometry (MS/MS) and revealed that a prototypical microbe, *Pyrococcus furiosus*, takes up 21 of 53 metals measured in its growth medium, 18 of which are present in macromolecular complexes. These results are in stark contrast to the five metals that had previously been identified in proteins individually purified from the same organism. Further, of the 343 metal peaks found across the fractions from a second level of chromatography fractionation (for the 10 metals that were detected), almost half (158) contained no known or predicted metalloprotein corresponding to that particular metal [6]. The purification of eight of these metal peaks resulted in the identification of novel metalloproteins, or proteins containing unexpected metal ions [6]. Unfortunately, this method has two major limitations. Firstly, given the large number (158) of unassigned metal peaks and difficulty in purifying a single protein, it is impractical to purify a significant portion of these novel metalloproteins. Secondly, it is not technically feasible to natively purify proteins of very low abundance over several chromatographic steps.

Herein is described a computational infrastructure and analytical methodology developed to both aid in the pursuit of novel metalloproteins [7] as well as to predict which proteins observed via MS/MS during this fractionation are likely to be metalloproteins without requiring purification to homogeneity. This required the development of a database, an Online Analytical Processing (OLAP) cube and InterPro-Metal (IPM) automated metal domain identification methods (directly supporting the pursuit of novel metalloproteins), as well as a Global Metal Protein Association (GMPA) analysis (enabling the prediction of metal-protein associations without complete purification). Given the essential biological role of metals, the discovery of novel metalloproteins has a multitude of implications in a variety of fields [1,8]. Moreover, the computational infrastructure and methods described can be applied to any form of biomass of interest from tissues to microbes to identify potential metalloprotein targets for experimental characterization. Most importantly, this analysis allows the discovery of low abundance metalloproteins without radioisotope labeling, which have eluded other methods [9,10] but which nonetheless may occupy key roles in essential biological pathways.

## Methods

### Experimental design

The experimental data set utilized in this study is an expanded version of the data set described in [7]. Briefly, native biomass of the hyperthermophilic archaeon *Pyrococcus furiosus *was fractionated anaerobically through multiple non-denaturing chromatography steps utilizing multiple column chemistries. The resulting 2,589 fractions were analyzed by ICP-MS to identify metals, and by MS/MS to identify proteins (primarily high-throughput MS/MS in this study). The MS/MS data were filtered such that the false discovery rate was less than 1%, as described in [7] and only proteins identified by Mascot with two or more peptides were considered in the current bioinformatics based study. The use of non-denaturing native chromatography, ICP-MS and MS/MS captures the co-occurrence of metals and proteins in their native form, and enabled a metal-based purification strategy, in contrast to conventional enzyme assay guided protein purification [6]. While this metal-based separation was used to purify a number of metalloproteins to homogeneity [6], the wealth of metal and protein data collected for proteins that were not explicitly targeted for direct purification provided an additional opportunity (applying data analysis techniques) to identify proteins that are likely to contain one or more metals in their native form.

### Data infrastructure

A relational database was constructed using Microsoft SQL Server 2005 to store the data used in this study. The database consists of three principal modules: a procedural (fractionation) module, a metal data (ICP-MS) module, and a protein data (MS/MS) module. The fractionation module was designed to store the procedural information used in each of the separation steps carried out during the multi-level column fractionation (allowing reconstruction of the complete experimental pathway). These multi-level hierarchical relationships between fractions were queried using recursive common table expressions (CTE). The metal data module was designed to store both procedural data and replicate metal concentration data for each sample and metal analyzed using ICPMS. This module also stores the peak assignments determined by manual inspection. Finally, the protein module stores data for each peptide identified by MS/MS, its protein source and the corresponding ORF, and details related to the MS/MS run and Mascot search as imported from Mascot XML result files. All fractions and samples (from fractions) collected were assigned unique IDs and labeled with 2 D data matrix barcodes to facilitate sample tracking. This ensured the simple and reliable association of the data contained in all three principal modules within the context of the experimental hierarchy.

**Figure 1:**
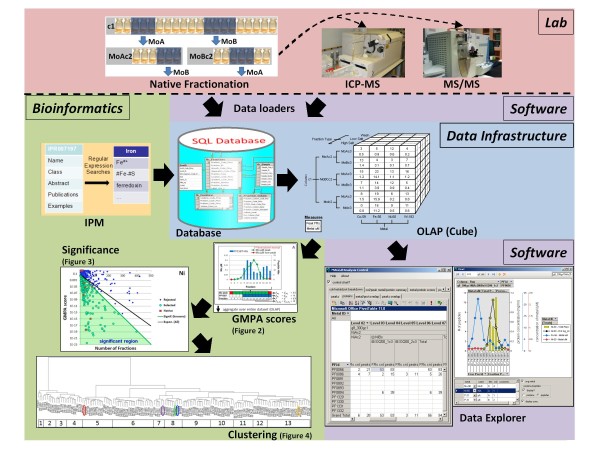
**Experimental and computational overview**. Below the dashed line is a schematic overview of the framework: all of the tools, databases and methods developed and utilized in this effort. Subsequent figures focus on the methods and calculations in the bioinformatics category.

The relational database contains 2,589 records corresponding to fractions (the fractionation module) obtained from the chromatographic separation in which 1,026 proteins (the protein module) were identified by MS/MS. Of these 1,026 proteins (corresponding to 135,989 peptide hit records in the database), 870 were identified with 2 or more peptides (125,777 peptide hit records) and were used in statistical scoring of metal association (GMPA scores, see below). Each of these fractions has associated metal concentration data generated by ICP-MS analysis for multiple metals depending in part on what metals were relevant to the goals of a given separation step (up to 53 with 78,514 overall metal concentration records; the metal module). An Online Analytical Processing (OLAP) cube (Figure 1, middle right) was constructed on top of the relational database using Microsoft SQL Server 2005's Analysis Services. The cube enabled efficient preprocessing of significant amounts of aggregate data (e.g. sums, averages, etc.) and at the same time enabled convenient data slicing across multiple dimensions of experimental data in real-time (e.g. queries as to the number of proteins and metals detected in the same fraction) [11]. OLAP is commonly used in the business analysis field but has not been widely exploited in scientific fields. However, OLAP is particularly well suited to the types of analyses presented herein given the hierarchical nature of the data set and the aggregate nature of queries utilized in this study to investigate experimental outcomes (e.g. are we purifying a protein-i.e. are metal/protein ratios improving?). A more detailed description of data infrastructure is given in the supplementary material (Additional Files 1, 2, 3, 4, 5). For the 870 proteins with GMPA scores, metal concentrations and numbers of peptides in the column fractions containing those proteins (designated by ORF number) are available at http://enigma.bmb.uga.edu/impact.

### Bioinformatic metalloprotein prediction-InterPro-Metal (IPM) automated metal domain analysis

The set of known metalloproteins that have been previously purified and characterized from *P. furiosus *by conventional chromatographic methods consists of only 23 proteins (encoded by 39 genes). Each contains one or more of Co, Fe, Ni, W and/or Zn atoms [6]. Although the utility of bioinformatic predictions is limited, such predictions can be used to identify homologs of more extensively characterized metalloproteins and serve as a starting point for assigning proteins to the observed metal peaks. The Integrated Resource of Protein Domains and Functional Sites (InterPro) [12] was used to predict known metal associated domains encoded in the genome of *P. furiosus*. InterPro integrates multiple popular protein feature databases, and provides the Iprscan utility for searching protein sequence queries against these databases. The genome was searched using this utility and the resulting matches of proteins to InterPro entries were stored in a relational database (protein-InterPro data). The description of each InterPro entry, including name, abstract and publication list, is available in a downloadable XML file ftp://ftp.ebi.ac.uk/pub/databases/interpro/interpro.xml.gz. This file was parsed and inserted into a corresponding relational database. A number of regular expression patterns relating to metal ions, metal cofactors and metal binding domains were used to search the text of each InterPro entry description to classify the entries which potentially involve specific metals (metal-InterPro data). Those metal-InterPro entries that had hits in the *P. furiosus *genome were evaluated manually for quality and assigned a subjective score. In some cases, keyword hits were not deemed to be indicative of a potential association of the given domain with a given metal, for instance an abstract for a particular subfamily of an enzyme may include additional information on other subfamilies which use alternate metals. Such spurious hits were assigned a score of 0 while metals with evidence of association with the given domain were assigned a positive score. All hits with nonzero scores were considered as potentially metal associated domains in subsequent analyses. The protein-InterPro data and metal-InterPro data were joined to determine which *P. furiosus *proteins had associations with specific metals and this subsequently will be referred to as the InterPro-Metal (IPM) database or InterPro-Metal analysis. These domain-based predictions were incorporated into the relational database and OLAP cube (Figure 1) to aid in the identification of novel metalloproteins and proteins for which the metal prediction and observed metal associations differ.

### Data driven metalloprotein prediction-Global Metal Protein Association (GMPA) analysis

**Figure 2:**
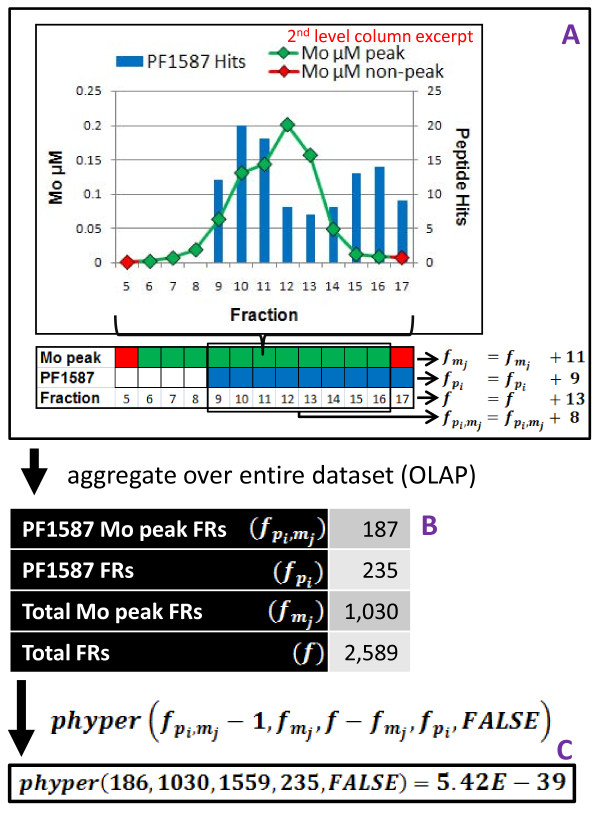
**GMPA score (Global Metal Protein Association) Calculation**. A, B and C illustrate the calculation with data for *p_i _*= PF1587 and *m_j _*= Mo, arrows represent generic steps in the calculation. A) Peptide counts for each protein (per fraction) are reduced to Boolean values (present/not present-shown as blue/white cells respectively). Whether a fraction is part of a metal peak or not is already a Boolean value (green/red cells). B) The "present/not present" values are counted across all fractions in the data set. C) The GMPA score is calculated from these values using the hypergeometric distribution and is roughly a p-value: how likely is a given protein to have been seen in metal peak fractions as many times as it was (or more) assuming an equal likelihood for the protein to have been observed in any fraction and given the number of metal peak fractions, protein fractions, and total fractions.

The previously described infrastructure enables efficient querying of the data along the following dimensions: protein identity, metal motif prediction for identified proteins, total protein concentration and metal concentration across the entire experimental data set. This database enables an evaluation of the global association of metals with proteins across the entire observed space. The heterogeneity of the utilized data, with ICP-MS yielding quantitative metal concentrations and the MS/MS results indicating the presence or absence of proteins (with peptide counts providing only local semi-quantitative comparison), dictated the use of methods which are less reliant on quantitative agreement between these data sets. Metal peak fraction regions were defined and entered into the database manually based roughly on the presence of at least two fractions in which the concentrations for a given metal (as indicated by two independent ICP-MS technical replicates) were substantially above the surrounding data for that metal. A hypergeometric distribution-based statistic that only considers the presence of proteins in these metal peaks instead of similarity of the shape or size of the metal and protein peaks (e.g. peptide counts of a given protein across a relatively contiguous set of fractions) was utilized. This statistic, the "Global Metal Protein Association score" (GMPA score, G(p_i_,m_j_)) is defined as:

$$\begin{array}{l} G(p_{i},m_{j})=\sum_{n=\max\left(f_{p_{i},m_{j}},f_{p_{i}}-(f-f_{m_{j}})\right)}^{\min(f_{p_{i}},f_{m}{}_{{}_{j}})}\frac{\left(\begin{array}{c} f_{m_{j}}\\ n \end{array}\right)\left(\begin{array}{c} f-f_{m_{j}}\\ f_{p_{i}}-n \end{array}\right)}{\left(\begin{array}{c} f\\ f_{p_{i}} \end{array}\right)}\\ =phyper\left(f_{p_{i},}{}_{m_{j}}-1,f_{m_{j}},f-f_{m_{j}},f_{p_{i}},FALSE\right) \end{array}$$

where *f *= the number of fractions in the data set, $f_{m_{j}}$ = the number of fractions inside of peaks of metal *j *in the data set, $f_{p_{i}}$ = the number of fractions in which protein *i *was observed, and $f_{p_{i,}m_{j}}$ = the number of fractions in which protein *i *was observed that are also contained within peaks of metal *j*. This statistic gives the probability of at least $f_{p_{i,}m_{j}}$ fractions out of the $f_{p_{i}}$ fractions in which protein *i *is found occurring within peaks of metal *j *assuming that the $f_{p_{i}}$ fractions were distributed randomly (with uniform probability) across the fraction space (Figure 2). The GMPA score was computed for all data sets we examined using the *phyper *function in R-2.10.1 [13] according to the formula above. The lower the score for a given protein/metal combination, the less likely it is that the protein and metal co-occur in the chromatographic fraction space by chance.

**Figure 3:**
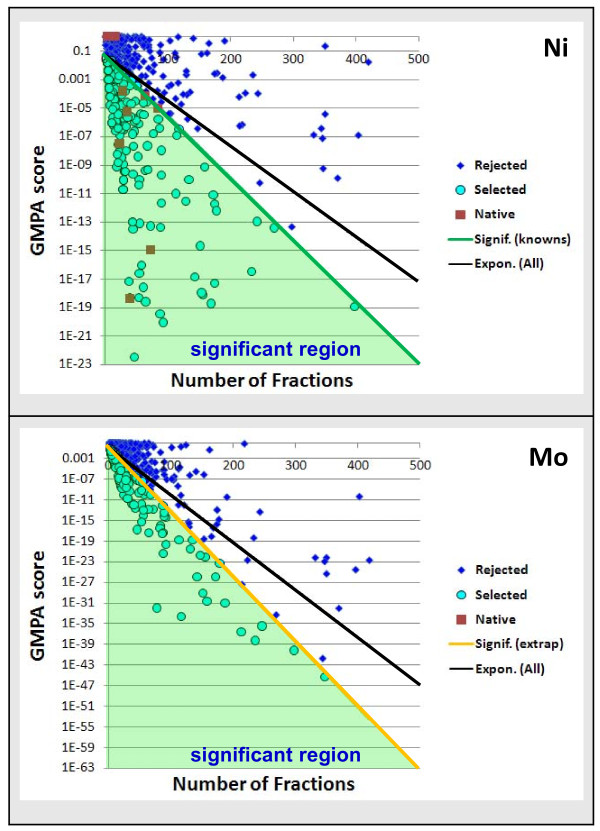
**Protein selection using GMPA score significance curve criterion**. Scatterplots (per metal) of the GMPA scores versus number of fraction occurrences for all proteins. The green/orange lines are exponential "significance" curves (plotted on a logarithmic scale) and proteins below are considered significant and selected for clustering (green regions and cyan points). The Ni significance curve (green) was based on occurrences of known Ni-proteins (red points) and the Mo significance curve (orange) was extrapolated from the relationships between the significance curves and exponential regression curves (through all points-the black lines) on average across all metals with known metalloproteins. Typically, this step removes an additional 20-35 proteins beyond what would be removed using the regression curves themselves.

The frequency of observation plays a large role in the GMPA score, so an exponential significance curve through the GMPA score/occurrences space was introduced dividing it into a set of proteins with substantial evidence for metal association (lower GMPA scores, fewer fraction occurrences) and a set without as much evidence (higher GMPA scores, more fraction occurrences). This provides an initial filtering step for our current real-world data set (Figure 3). For metals significantly represented in the set of known metalloproteins (Fe, Ni, and W), the significance curve was generally set using these known references. For metals present in few or no known metalloprotein(s), the curve was extrapolated from the sets with sufficient known metalloproteins (Additional File 6). This extrapolation was based on the ratios between the exponent of an exponential regression curve calculated over all proteins through the GMPA score/occurrences space (Figure 3) and the significance curves chosen to capture all known metalloproteins that could reasonably be captured for Fe, Ni and W. Typically, an absolute GMPA score cut-off was employed after significance curve filtering to remove proteins that occurred in fewer than approximately 10 fractions from further analysis since it is unlikely that anything can be determined from our data with any confidence for such proteins.

**Figure 4:**
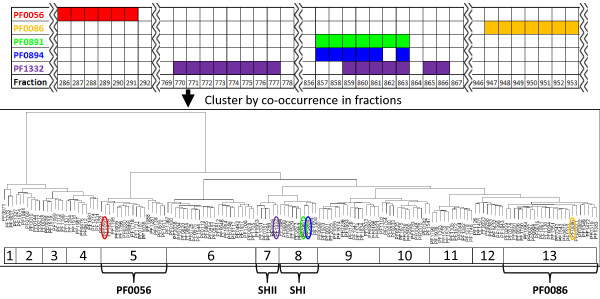
**Hierarchical clustering of GMPA score criterion significant nickel associated proteins**. Potential nickel proteins clustered based on co-occurrence in fractions using Ward's method. The grid at top contains excerpts from the data set selected to give a rough sense of protein co-occurrence within fractions and its effect on the resulting clustering. The numbered boxes at the bottom indicate the partitioning of the overall clustering into self-contained clusters as determined by cutreeHybrid (indicated by colors in supplementary material clusterings). Clusters containing known Ni-proteins are highlighted at the bottom illustrating the clustering together of subunits of known nickel-proteins and clustering apart of distinct Ni-proteins (SHI: soluble hydrogenase I PF0891-PF0894, SHII: soluble hydrogenase II PF1329-PF1332).

To further organize the space of potential metalloproteins and to estimate the number of metalloproteins that could reasonably be expected from among the set of GMPA score filtered proteins for each metal, hierarchical clustering was applied. Hierarchical clustering organizes the elements of a set into a tree based on similarity (co-occurrence of proteins in fractions in this case). The resulting tree can facilitate the partitioning of the original set into sub-tree "clusters" capturing natural divisions in the data. This technique is particularly useful when the number of natural groups that might exist in the data is not known a priori. Ward's method of hierarchical clustering, which is variance-minimizing, along with the Euclidean metric and the Dynamic Tree Cut package for R [14] were found to give useful self-contained clusters. The cutreeHybrid function of the Dynamic Tree Cut package was used to analyze the trees generated for each metal (Figure 4, Additional Files 7 and 8-graphics produced in part using slightly modified versions of functions from [15]). The parameters passed to this function were tuned for Mo and Ni so that within the selected clusters there were clear "core" regions (composed of fractions with ≥50% of proteins in the cluster observed) while "core" region overlap was minimal between pairs of clusters. Low overlap between clusters in metal peak regions, with which core regions typically coincide, makes it likely that each cluster should contain at least one metalloprotein in order to explain the metal peak data-i.e. that the dimensionality reduction achieved in the clusterings is reasonable. Approximate minima required to cover all peaks were also calculated using the greedy algorithm approach to the set cover problem [16] and were found to be consistent with the results of clustering. The parameters found for Mo and Ni were then applied for all metals.

## Results and Discussion

The computational framework that was developed consisted of a database, an Online Analytical Processing (OLAP) cube, InterPro-Metal (IPM) automated metal domain identification and Global Metal Protein Association (GMPA) analysis. This complemented and enhanced our recent effort to elucidate the metalloproteome of *P. furiosus *and to identify novel metalloproteins [6]. The GMPA analysis in particular was used to provide estimates of the numbers of metalloproteins that could be expected proteome-wide and a narrowed list of candidates (based on ICP-MS and MS/MS data) at various stages of the column chromatography fractionation, culminating in our predictions at the conclusion of the study that the numbers of undiscovered metal containing proteins in *P. furiosus *range from approximately 5 for vanadium up to as many as 13 for cobalt. Validation of these predictions is provided by the overall success of the GMPA analysis in categorizing known metalloproteins from *P. furiosus*, the establishment of lower bounds on the numbers of proteins required to explain all metal peaks seen during the fractionation, the fact that the predictions lie within reasonable ranges in the context of literature (where up to a third of proteins are expected to contain or be involved with metals in various ways with the caveat that the majority are likely to be involved with Mg) [2,17,18] and considering the effect of dynamic association/adventitious metal binding [19].

In order to determine how much of the information contained in our data set remains uncaptured by the GMPA analysis, nickel (Ni) and molybdenum (Mo) were chosen for manual evaluation using the GMPA predictions as a starting point. Of the 870 proteins identified by MS/MS with two or more peptides, 153 and 119 were found to be significantly associated with Ni and Mo respectively, upon clustering yielding predictions of 13 and 10 total Ni- and Mo-proteins in the proteome of *P. furiosus*. The local semi-quantitative MS/MS peptide hit data for each of the proteins clustered was then manually compared to the local metal concentration data using our data explorer (Figure 1). This step excluded an additional 131 and 99 proteins producing top candidates lists of 22 and 20 proteins that are most likely to contain Ni and Mo respectively. These lists were then analyzed more extensively through bioinformatic analyses and literature searches. We will first describe the results obtained at the conclusion of our experimental study and then discuss the bioinformatics of the lists of predicted Ni- and Mo-proteins, concluding with the limitations inherent in this study.

### Bioinformatic metalloprotein prediction-InterPro-Metal (IPM) automated metal domain analysis predictions

**Figure 5:**
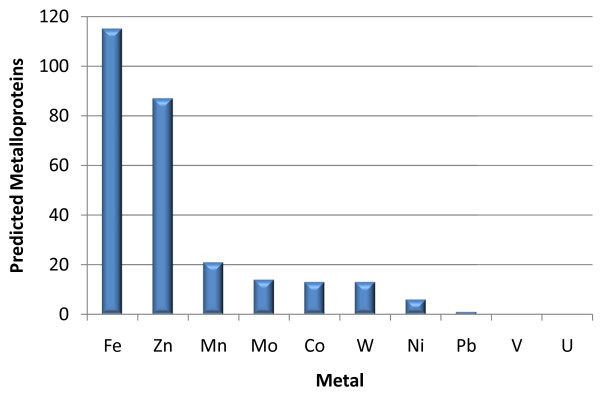
**IPM predicted metalloproteins identified by MS/MS**. Out of the 870 proteins identified with two or more peptides, 221 were predicted to be metalloproteins. The majority were predicted to contain Fe or Zn with fewer predicted to contain Mn, Mo, Co, W and Ni.

Of the 2,065 annotated opening reading frames (ORFs) in the RefSeq annotation of the *P. furiosus *genome [20], 376 were found to have matches to metal-associated InterPro entries. These included all of the 23 previously known metalloproteins [6]. Of the 376, 221 were detected by MS/MS with 2 or more peptides, 43 of which had matches to multiple metals, either from matches to multiple InterPro entries, or to a single InterPro entry that lists potential associations with multiple metals. Consistent with expectation, the majority of the *P. furiosus *proteins with metal-related InterPro hits were predicted to be associated with Fe or Zn, with fewer Mn, Mo, Co, W and Ni predictions (Figure 5) [4,17]. There were no predictions of association with lead or uranium other than transport proteins whose InterPro descriptions may list many metal ions. The observation of a metal peak in a fraction in which no predicted metalloprotein was identified shows that one of the proteins identified is a completely novel metalloprotein, or one which uses a metal other than that expected by its annotation [6]. An unexpectedly large number-158 of the 353 metal peaks detected for the second level of column fractionation-were found in the data set.

### Data driven metalloprotein prediction-Global Metal Protein Association (GMPA) analysis predictions

The 870 proteins identified by MS/MS with two or more peptides were assigned GMPA scores and partitioned into significant and insignificant regions as described in the methodology section. The number of proteins deemed significant ranged from 45 for V to 153 for Ni (Table 1). The proteins falling in the significant regions for each metal were then hierarchically clustered by co-occurrence in fractions, with the number of clusters ranging from 5 for V to 16 for Co (Table 1). Each of these clusters is assumed to contain at least one metalloprotein giving rise to the observed metal peaks (see Methods). A total of 23 metalloproteins are known for *P. furiosus *from previous studies [6], and the coverage of the corresponding clusterings by these standards, together with the metalloproteins discovered by metal-directed purification [6], is summarized in Table 1. As a specific example of the data underlying each row of this table, Figure 4 shows the 13 clusters into which the 153 significant (by GMPA score) Ni targets fell. The two known Ni-containing enzymes, the soluble hydrogenases, lie in distinct clusters, as illustrated in Figure 4.

**Table 1:** GMPA Analysis Clustering and Coverage of Known Metalloproteins

Metal	Known metalloprotein subunits			Proteins Clustered	Clusters	
	
	Total	Observed	Met GMPA significance criterion		Total	With known metalloproteins
	
Co	5	5	3	139	16	3
**Fe**	35(20)	35	19	148	15	8

**Mn**	0	0	0	73	7	0

**Mo**	2	2	1	119	10	1

**Ni**	12(5)	12	9	153	13	4

**Pb**	0	0	0	90	9	0

**U**	0	0	0	76	7	0

**V**	0	0	0	45	5	0

**W**	5	5	4	136	11	4

**Zn**	5	5	1	116	9	1

Some proteins consist of multiple subunits, the number of holoenzymes are given in parentheses. Some proteins contain multiple metals and factor into multiple rows. For full clusters, refer to supplementary material.

### Nickel- and Molybdo-protein evaluation

Nickel and molybdenum were selected for further detailed analysis primarily using a data explorer developed in-house leveraging the speed of the OLAP cube (Figure 1). There are 12 genes that encode subunits of known Ni-containing proteins in *P. furiosus*. A total of seven of the 12 proteins encoded by these genes were detected by MS/MS analyses including five genes that encode the two multi-subunit Ni-containing soluble hydrogenases (I and II) of *P. furiosus*. These seven genes represent a set of positive controls for evaluation of the analysis. Prior to the metal-targeted comprehensive protein purification [6], *P. furiosus *was not known to have any Mo-proteins, so all proteins identified by that analysis represent novel Mo-proteins in this organism. For Ni, out of 870 proteins observed by MS/MS in at least one fraction of the fraction-space used for this analysis, 153 proteins were selected in the initial GMPA score significance curve screening. The parameters for the significance curve for Ni were chosen directly based on the known Ni-proteins such that the significance curve did not filter out any of the subunits of the Ni-containing soluble hydrogenase I (PF0891-PF0894) and only filtered out one of the subunits of the Ni-containing soluble hydrogenase II (PF1330). This is essentially unavoidable given that PF1330 was found in relatively few (16) fractions. From the 153 proteins, 13 reasonably distinct clusters were defined after hierarchical clustering and each was manually evaluated (Table 2, Additional File 9). Seven of the clusters were found to contain proteins exhibiting local agreement of MS/MS data to the Ni ICP-MS data and 22 proteins or subunits of proteins were found in total (Table 2). Five of the 22 best candidates for Ni are in fact subunits of previously known Ni-protein complexes (Table 2) providing validation of this approach. An additional 2 of the 22 candidate Ni-proteins were very recently taken to purity or partial purity (PF0056 and PF0086) by the metal-directed purification and their assignment was confirmed [6]. This leaves 15 potential novel Ni-containing proteins on which to carry out further experiments.

**Table 2:** Manually Evaluated Nickel Protein Candidates

Cluster number	ORF	Annotation	Crystal structure homolog	Metal In structure	IPM prediction
2	PF0144	Aldolase-type TIM barrel			Fe

2	PF1881	Alba archaeal DNA/RNA-binding protein	2Z7C		

3	PF0038	Beta-lactamase-like glyoxalase II family member			Zn

4	PF1916	Glycosyl transferase, family 2			

4	PF1987	Conserved hypothetical protein			

***5***	***PF0056***	***Carbohydrate binding protein***	1VJ2	***Mn***	***Mn***

5	PF0138	Uncharacterized rubrerythrin domain protein	2FZF		Fe

6	PF1664	Phosphoribosyl-AMP cyclohydrolase	1ZPS	Cd	Zn

6	PF2038	Adenosylcobalamin biosynthesis	1G5T	Mg	Co

**7**	**PF1329**	**Hydrogenase II beta**			**Fe**

**7**	**PF1331**	**Hydrogenase II delta**			**Fe**

**7**	**PF1332**	**Hydrogenase II alpha**			**Fe,Ni**

**8**	**PF0891**	**Hydrogenase I beta**			**Fe**

**8**	**PF0894**	**Hydrogenase I alpha**			**Fe,Ni**

8	PF1500	PRC-barrel-like			
8	PF1529	Pyroxidine biosynthesis protein	2YZR		

12	PF1401	Peptidyl-prolyl cis-trans isomerase			

***13***	***PF0086***	***Alanyl-tRNA synthetase, class IIc***	2E1B	***Zn***	

13	PF0615	Hydrogenase expression/formation protein A	3A43	Zn	Ni

13	PF1272	LamB/YcsF	1V6T		

13	PF1684	Acetylglutamate kinase	2EGX		

13	PF1861	Lysyl aminopeptidase	2PE3		Zn

Cluster number refers to hierarchical clustering with dynamic hybrid partitioning, see Figure 4 for explanation and supplementary tables 1-8 in Additional File 9 for complete cluster tables. Crystal structures obtained from PDB with sequence similarity >50%. ORF numbers in bold have been previously characterized in *P. furiosus*, those in italics were characterized by metal-targeted purification.

For Mo, out of 870 proteins observed by MS/MS in at least one fraction of the fraction-space used for this analysis, 119 proteins were selected in the initial GMPA score significance curve screening. The significance curve for Mo was selected (as described in Methods) by extrapolation based on the significance curves chosen for metals with known metalloproteins. From the 119 proteins, 10 clusters were defined after hierarchical clustering and each cluster was manually evaluated. Six of the clusters were found to contain proteins exhibiting local agreement of MS/MS data to the Mo ICP-MS data and 12 such proteins were found in total (Table 3, Additional File 9). A novel Mo-protein (PF1587) was purified by the metal-based chromatography method from which the data set was derived [6] and this is identified by the GMPA analysis as a likely Mo-protein. This nicely demonstrates the effectiveness of the method given sufficient observation. Finally, comparing the results of the IPM analysis to the manually evaluated GMPA analysis derived results, among the 22 manually evaluated targets deemed to be likely Ni-proteins, only 3 had a Ni IPM match with 10 additional proteins having Co, Fe, Mn and Zn matches. Of the 20 manually evaluated targets deemed to be likely Mo-proteins, only 1 had a Mo IPM match.

**Table 3:** Manually Evaluated Molybdenum Protein Candidates

Cluster	ORF	Annotation	Crystal structure homolog	Metal in structure	IPM Metal
1	PF0009	ThiF family protein	1JWB	Zn	

1	PF0187	Putative cofactor synthesis protein			Fe,Mo,W

1	PF0668	YjgF-like protein	2DYY		

1	PF1718	Wyosine base formation, Radical SAM	2YX0		Fe

1	PF1766	Cell division transporter FtsY	3DMD		

2	PF1956	Fructose-1,6-bisphosphate aldolase class I	1OJX		

3	PF1828	Protein of unknown function DUF1621			

3	PF1886	Carbohydrate/purine kinase			

4	PF0098	NAD+ synthase	2E18		

4	PF0236	Phosphoribosyl pyrophosphokinase	1U9Y		Mg

4	PF1401	Peptidyl-prolyl cis-trans isomerase	1IX5		

4	PF1675	Asp/Glu/hydantoin racemase	2ZSK		

4	PF1731	Signal recognition particle 54	3DM5		

5	PF1538	Amidohydrolase 1	1P1M	Ni	

7	PF0523	Protein of unknown function DUF509	1ZD0	Mg	

7	PF1222	Protein of unknown function DUF217			

***8***	***PF1587***	***Protein of unknown function DUF89***	2G8L		

9	PF0212	DNA polymerase, family B	2JGU	Mn	

9	PF0306	Translation factor, SUA5 type			

9	PF0463	Phosphoglycolate phosphatase			

Cluster number refers to hierarchical clustering with dynamic hybrid partitioning, see Figure 4 for explanation and supplementary tables 1-8 in Additional File 9 for complete cluster tables. Crystal structures obtained from PDB with sequence similarity >50%. ORF numbers in bold have been previously characterized in *P. furiosus*, those in italics were characterized by metal-targeted purification.

### Bioinformatic analyses of predicted Ni- and Mo-proteins

As discussed above, 7 of the 22 genes listed in Table 2 encode proteins or subunits of proteins which have been shown to contain Ni ions in *P. furiosus*. This includes subunits of soluble hydrogenase I (SHI) and soluble hydrogenase 2 (SHII) grouped in clusters 7 and 8 respectively. In addition, PF0615 in cluster 13 is annotated as a hypA protein, which is implicated in Ni insertion in the hydrogenases. The structure of a hypA homolog from *Thermococcus kodakaraensis *has been solved and its Ni-binding site described [21]. This demonstrates that cluster 13 has at least two Ni-binding proteins that frequently co-occur in the fractionation space. Of the five ORFs in Table 2 with homologs whose crystal structures have been solved bound to metals other than Ni, three are now known to bind Ni (PF0056 and PF0086) in *P. furiosus *or are known to have a Ni-binding site (PF0615). In particular PF0086 has been shown to bind Ni [6], but its homolog from the closely related *Pyrococcus horikoshii *(PDB 2E18) was expressed recombinantly and crystallized in a Zn-bound form. This illustrates the flexibility of metal binding domains [22], and their ability to bind biologically incorrect metals when expressed heterologously [23]. The two remaining ORFs with non-Ni homolog crystal structures are PF1664, which contains the cysteines that bind Zn (Cd in the crystal) in its homolog [24] and may be involved in binding Ni in *P. furiosus*, and PF2038 with a homolog that binds Mg-ATP. The only protein listed in Table 2 that is likely not to contain Ni is PF1861. This was previously purified from *P. furiosus *biomass and contained Co and Zn but not Ni [25]. This leaves 13 proteins that are predicted to contain Ni. These proteins have no known or conjecturable Ni associations based on their sequences and are assumed to predominantly contain a set of undiscovered Ni-binding sites. Finally, it is worth pointing out that PF0056 is one of five ORFs (PF0144, PF1987, PF0056, PF0138 and PF1500) annotated as either conserved hypothetical or with only domain/motif matches and has now been shown to bind Ni [6].

In contrast to the case of Ni, the pool of known molybdo-proteins in *P. furiosus *is small and far less can be ascertained bioinformatically. In particular, the role of Mo-proteins in *P. furiosus *is unclear, with only two such metalloproteins having recently been identified [6]. Consequently, only two of 20 proteins in Table 3 have either been shown to bind Mo (PF1587) or have an IPM hit for Mo (PF0187). The other recently identified Mo-protein that was purified from *P. furiosus *(PF1972) was observed in only 17 chromatography fractions and was rejected by the GMPA analysis, which depends on sufficient levels of occurrence in the data set to establish significance of metal-protein association. On the positive side, many of the uncharacterized proteins contain residues that could be involved in Mo-binding (e.g. 14 of 20 contain at least one cysteine residue as is often involved in Mo-pterin binding) [26-28], but given the extent and complexity of typical molybdopterin binding interactions and biochemistry [29] and the lack of knowledge of Mo-binding in organisms closely related to *P. furiosus*, we have not looked into this aspect further. On the negative side, DNA polymerase (Mo cluster 9, Table 3), which has been well studied in many different organisms and is not known to bind or utilize Mo (although it is not clear if this has been directly considered previously) was picked as a top candidate Mo-protein. This illustrates that some of the targets that appear to reliably co-occur with a metal may be coincidental, or the result of interaction natively with additional proteins that are not strictly required for their primary function. Interestingly, four of the 20 predicted Mo-protein candidates have annotations that include "domain of unknown function." The confirmation of Mo-binding, which has already occurred for PF1587 by metal-directed purification, should provide an improved foundation for functionally characterizing these conserved domains which so far have been elusive [6].

### Known limitations

We initially attempted to use standard correlation-based statistical techniques such as principal component analysis (PCA) and canonical correlation analysis (CCA) to determine associations between metals and proteins based on the experimental data that were available [6]. However, these efforts were hindered by the relatively non-quantitative nature of the MS/MS data available (lacking even spectral count information). Consequently, the GMPA analysis method was developed which is less reliant on quantitative agreement between these data sets. Simulated data sets demonstrated the effectiveness of GMPA scores alone given adequate separation regardless of the amount of noise observed in the peptide counts, but it was discovered that the metal-based fractionation typically did not produce a comprehensive enough data set containing an appropriate degree of overall separation. For example, the experimental data set is most consistent and comprehensive at the second column level (termed C2 in [7]) and separation is still relatively incomplete at this level. Consequently significance cut-off curves and clustering were employed completing the overall GMPA analysis methodology. It is likely that the predictive power of the methodology could be greatly improved by utilizing a data set with a more comprehensive fractionation across all levels, through the use of more quantitative MS/MS techniques [30-32] and more powerful statistical techniques (PCA/CCA) that could then be applied more easily. This methodology could also potentially be carried out in a more automated fashion on an analytical scale to provide a rapid determination of the metalloproteins of any organism.

## Conclusions

We have presented a computational methodology that can uncover probable metal-containing proteins using data from a non-comprehensive native fractionation coupled with metal and protein measurement using ICP-MS and MS/MS. This methodology has identified a number of candidate novel metalloproteins that are targets for future experimental verification. Application of the method to simulated data sets indicates that additional predictive accuracy could be achieved through the use of a more comprehensive fractionation. Our results for each of the 10 metals examined in this study underscore the unexplored complexity of metalloproteomes and have broad implications for protein structure and function as well as metal toxicity.

## Abbreviations

The abbreviations used are: Co: cobalt; Fe: iron; Mn: manganese; Mo: molybdenum; Ni: nickel; Pb: lead; U: uranium; V: vanadium; W: tungsten; Zn: zinc; HT-MS/MS: high-throughput tandem mass spectrometry; ICP-MS: inductively coupled plasma mass spectrometry; ID: identifier; MS/MS: tandem mass spectrometry; OLAP: online analytical processing; ORF: open reading frame.

## Authors' contributions

WAL developed the bioinformatic metalloprotein prediction approach. JLP developed the OLAP infrastructure, data explorer software and Global Metal Protein Association analysis approach and manually evaluated the Mo and Ni clusters. WAL and JLP performed and interpreted the results of bioinformatics analyses of manually evaluated candidates. FLP, WAL and JLP developed the database infrastructure. JLP, FLP and WAL developed the ENIGMA IMPACT website. FLP and JLP wrote data loaders and loaded MS/MS and chromatographic fractionation data into the database. FLP and JLP wrote ICP-MS data loading software and AC loaded all ICP-MS data and defined all metal peaks manually using this software. AC performed ICP-MS analyses. SAT, EK, JVA and GS performed HT-MS/MS analyses. AC, ALM, MPT and JWS grew and fractionated *P. furiosus*. AC, ALM, FEJ, FLP, JWS, MPT and JAT and MWWA developed the original metal-directed purification. JLP, MWWA and WAL wrote the manuscript. All authors read and approved the final manuscript.

## Supplementary Material

Additional file 1 **Data infrastructure additional details**. A more detailed description of the data infrastructure. Click here for file

Additional file 2 **Relational database schema figure**. A diagram illustrating the basic layout of the relational database. Click here for file

Additional file 3 **OLAP cube figure**. A diagram illustrating the basic connections between measure groups and dimensions in the OLAP cube. Click here for file

Additional file 4 **Relational database schema description**. Click here for file

Additional file 5 **OLAP schema**. The complete schema for the OLAP cube as an XML based backup. Click here for file

Additional file 6 **Significance curves, function calls for clustering**. Significance curves and parameters used to generate all clusterings, clusters. Click here for file

Additional file 7 **Cluster Diagrams**. Tree diagrams of clusters. The colors are used simply to distinguish the defined clusters. Click here for file

Additional file 8 **Cluster Tables**. Supplementary tables 1-8 containing complete cluster details. Click here for file

Additional file 9 **GMPA scores/occurrences tables**. GMPA scores for all metals and proteins studied. The number of fraction occurrences in the data set is listed for each protein as well. Click here for file
