# Supplementary material for: A Computational Framework for Proteome-Wide Pursuit and Prediction of Metalloproteins using ICP-MS and MS/MS Data
Source: BMC Bioinformatics. 2011 Feb 28;12:64. doi: 10.1186/1471-2105-12-64 (PMC3058030; doi:10.1186/1471-2105-12-64)
Supplement: Additional file 1 — Data infrastructure additional details. A more detailed description of the data infrastructure. [file 1471-2105-12-64-S1.DOC]

Description of Data Infrastructure

A relational database was constructed to store procedural data and experimental observations generated by the multilevel (hierarchical) chromatographic fractionation experiments. This database, named ExpressM3, was constructed using Microsoft SQL Server 2005 and consists principally of three modules: a procedural module which contains details of the biomass fractionation, an ICP-MS/metal data module, and an MS/MS protein data module. The procedural module was designed to store the protocol information used in each of the separation steps, intermediary concentration steps, and other steps carried out during multi-level column fractionation. The ICP-MS data module was designed to store data generated by multiple technical replicate ICP-MS analysis of material generated along with the parameters used in each run of the inductively coupled plasma mass spectrometer. The ICP-MS module was also designed to store metal peak information so that the base criteria used to define metal peaks could be modified with reference to previous assignments and the results of such assignments vetted by human experts. Finally, the MS/MS module was designed based on the structure of the XML files generated by Mascot during protein sequence database searches as described later. All data were linked through the tb_Fraction and tb_Sample tables containing records tied directly to physical fractions collected during the multi-level column-based separation and samples taken of these fractions for ICP-MS, MS/MS or other analyses.

The procedural module consists of two submodules, one concerned with capturing the protocol related details used in separations and the other concerned with cataloguing and tracking the materials generated and their flow through the system. The protocol submodule contains the tb_Protocol table with relatively generic information (e.g. a basic description of project specific methods for running a given type of column) keyed to by tables more specific to a given separation or class of separations, tb_Separation and tb_Separation_Class. For example, while many of the parameters used for a gel filtration were common across all such gel filtration steps, the type of buffer used, its concentration and other details did change for certain groups of samples. Even more variation is typical with parameters such as the quantity of sample loaded onto a column and details such as the specific AKTA system on which a sample was run. The segmentation of this information into tb_Protocol, tb_Separation_Class, tb_Separation, and tb_Gradient (related to chromatography column gradients) appropriately models the underlying data to be captured (see Additional file 6 for figure and Additional file 8 for schema). The materials tracking submodule consists principally of the tb_Fraction, tb_Sample and tb_Combine tables. The autoincrementing integer primary keys of the tb_Fraction and tb_Sample tables serve not only to tie together much of the rest of the information within the database but also serve as the actual IDs (appended to the prefixes FR- for fractions and SA- for samples) used to label physical materials for storage (as printed onto labels utilizing 2D Data Matrix barcodes). The tb_Fraction table uses self-referencing to represent and preserve the fractionation hierarchy wherein the fractions and fraction pools from a given fractionation level are linked to the parent of that level. This creates a hierarchy that is particularly straightforward to traverse when the details of any particular step are of interest. Finally, the tb_Combine table tracks which specific fractions from a given parent node were pooled (e.g. consecutive metal peak fractions) for loading onto a successive columns for further fractionation (i.e. during purification of a particular metalloprotein).

The ICP-MS and MS/MS modules store metal (or more generally element or isotope) and protein data obtained through application of these mass spectrometry based techniques to samples collected from fractions created during chromatography based fractionation. The MS/MS module is particularly straightforward as it simply stores the data generated by Mascot upon searching the MS/MS data collected for a specific sample. This data is linked through sample identifiers that ultimately link to fraction identifiers (tb_Fraction). The ICP-MS module is somewhat more structured as it was specifically set up to capture technical replicates. Consequently, the tb_ICPMS_Dataset table contains records corresponding to a specific column in the fractionation hierarchy (and the fractions off of this column) and the tb_ICPMS_Run table contains records corresponding to technical replicates (i.e. multiple runs of the same samples) on the ICP-MS instrument. Other tables hold metal concentrations (tb_ICPMS_Data), parameters used in each run (tb_ICPMS_Parameter) and metal peak definitions (tb_ICPMS_Peak and tb_ICPMS_Peak_Data - one defining a group or peak, the other defining the actual samples/fractions within the peak).

Challenges were encountered in ensuring that the database remained up to date with respect to experiments and measurements that had been run. Further, analyzing and accessing the data collected also presented a challenge. On the input side, a variety of technologies were used in an attempt to find a balance that would not be too taxing to either the bioinformaticians or the experimentalists. Ultimately, procedural data was submitted by experimentalists through a simple Microsoft Excel template built to reflect key tables of the procedural module (tb_Protocol, tb_Separation_Class, tb_Separation, tb_Fraction, tb_Sample) and designed to be easy to fill in through the use of hidden tables that could easily be kept up to date with respect to the database (containing, for example, references to previously specified protocols). The maintenance of this template with respect to the state of the database occurred through the use of software written using "Visual Studio Tools for Office" (VSTO) and Visual Studio 2008. Parsing and uploading of the data contained in these sheets was carried out using software written with VSTO as well. ICP-MS data was similarly uploaded using an Excel worksheet that read comma separated value (csv) output from the ICP-MS instrument software and converted it to a form suitable for our database. In this case VBA was utilized. The MS/MS data was loaded into the ExpressM3 database using a Microsoft Visual Basic 6.0 application specifically written to validate and parse Mascot XML search result files directly into a relational database. For output, Excel was primarily used to connect to and display data from an OLAP data source via pivot tables for the work discussed in this manuscript.

The Online Analytical Processing (OLAP) cube (Figure 1, middle right, Additional file 7 for figure and Additional file 9 for schema) was constructed on top of the relational database. In the field of data management, relational databases are commonly called transactional data stores. This is due to the fact that a relational database ensures consistency and efficient (concise) storage and input of data (transactions) but is not typically well suited to flexible retrieval and analysis of that data. OLAP (analytical) technologies were developed to address this need. OLAP provides efficient preprocessing of aggregate data and enables convenient data slicing across multiple dimensions of data pulled from a transactional database. This layering/separation allows for the use of systems ideally suited for accurate and efficient recording of data (transactional) while separately supporting the needs of analysts and bioinformaticians for flexible access and display of the data collected. For example, the OLAP cube was utilized to present averaged ICP-MS metal micromolar concentrations (average over individual series stored directly in the database from technical replicates), to immediately display which column fractionations contained a particular protein, to calculate the overlap of metal peaks and specific proteins as identified by mass spectrometry, as well as in many other data display roles. Once a specific calculation is specified in an OLAP project, the OLAP software will apply that calculation to all data in the database upon recompilation automatically precomputing many values of interest. This substantially improves the experience for the data analyst who will often frequently have interesting ideas during the course of an analysis that can typically be checked easily through an appropriate data presentation. With only a transactional database, it might take many minutes to run calculations over all of the data to validate or discard a particular idea whereas it is often possible to slice the data in real-time using a precompiled OLAP cube in such a way as to immediately confirm or refute a hypothesis.The options we have chosen for the OLAP cube might be considered by any laboratory running similar experiments. We have not attempted to tailor any of our infrastructure for more general use as it was created within the framework of a specific project and was not the primary focus of our work. However, we welcome inquiries from bioinformaticians interested in our infrastructure and our experience in building it.
