# Supplementary figures and images for: A Computational Framework for Proteome-Wide Pursuit and Prediction of Metalloproteins using ICP-MS and MS/MS Data
Source: BMC Bioinformatics. 2011 Feb 28;12:64. doi: 10.1186/1471-2105-12-64 (PMC3058030; doi:10.1186/1471-2105-12-64)

## Slide 1
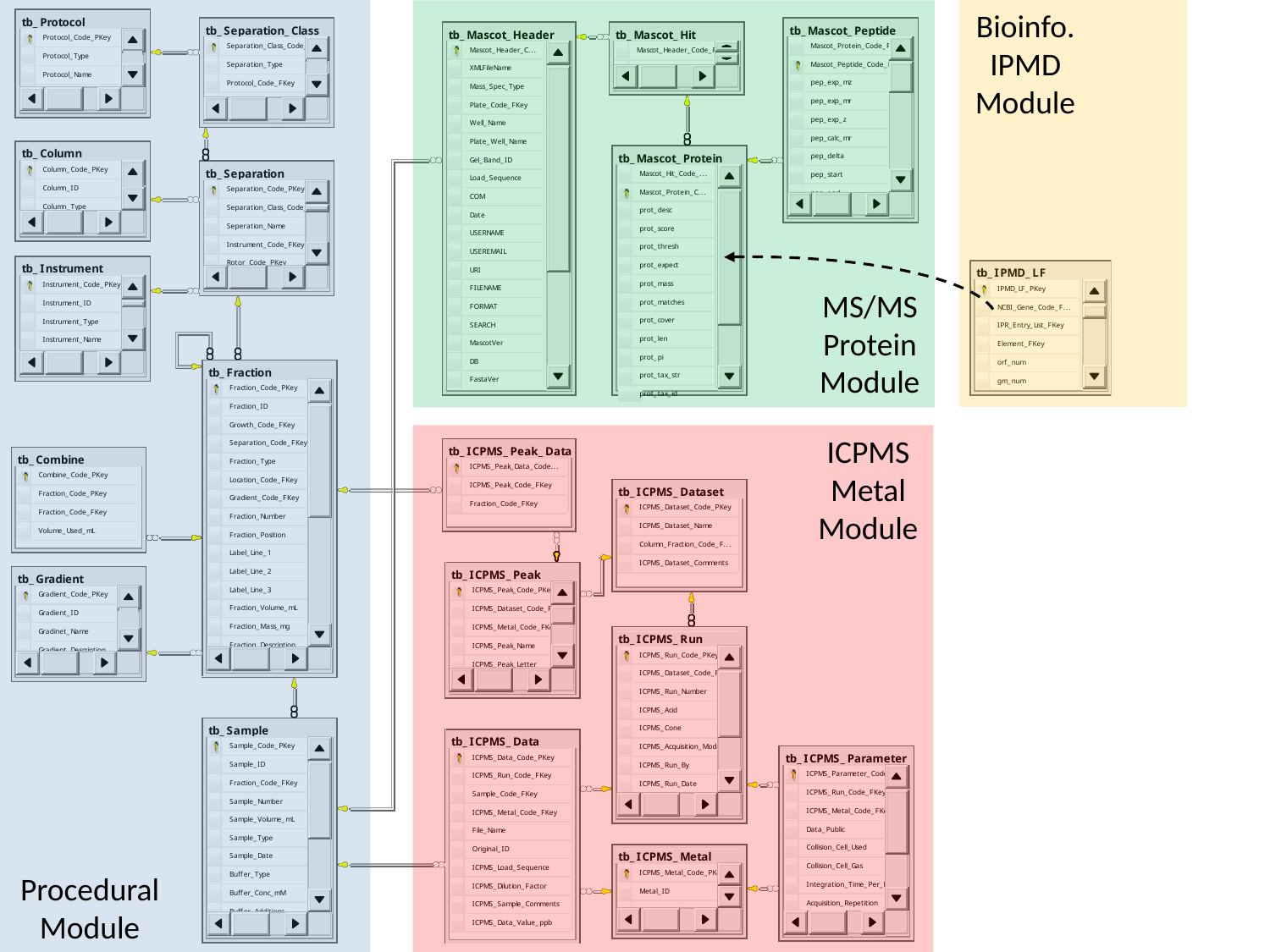

Bioinfo.IPMD Module
MS/MS Protein Module
ICPMS
Metal Module
Procedural Module

Supplement: Additional file 2 — Relational database schema figure. A diagram illustrating the basic layout of the relational database. [file 1471-2105-12-64-S2.PPTX]

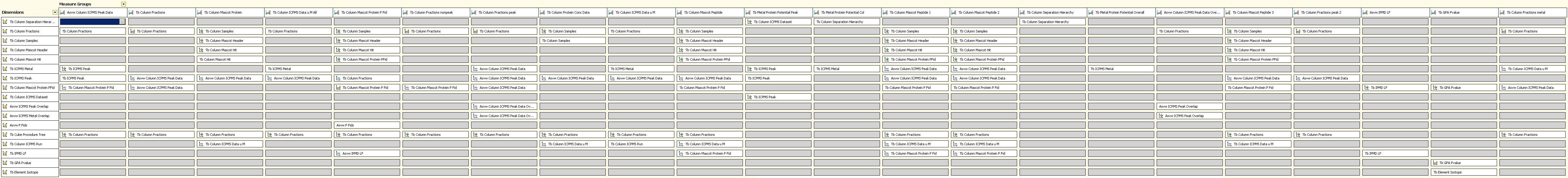

Supplement: Additional file 3 — OLAP cube figure. A diagram illustrating the basic connections between measure groups and dimensions in the OLAP cube. [file 1471-2105-12-64-S3.PNG]
