# Supplementary material for: A Computational Framework for Proteome-Wide Pursuit and Prediction of Metalloproteins using ICP-MS and MS/MS Data
Source: BMC Bioinformatics. 2011 Feb 28;12:64. doi: 10.1186/1471-2105-12-64 (PMC3058030; doi:10.1186/1471-2105-12-64)
Supplement: Additional file 6 — Significance curves, function calls for clustering. Significance curves and parameters used to generate all clusterings, clusters. [file 1471-2105-12-64-S6.PPT]

## Slide 1
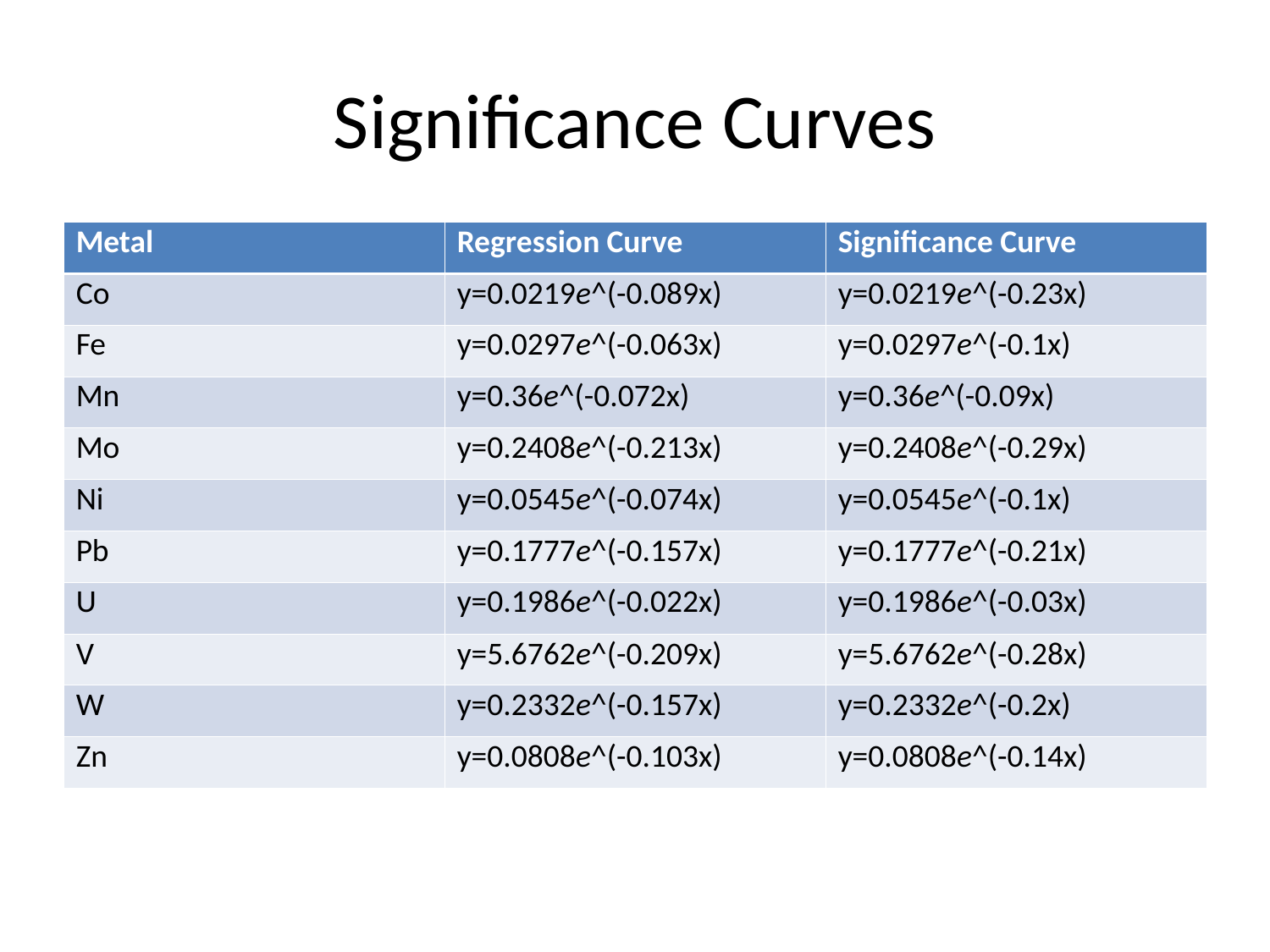

# Significance Curves
| Metal | Regression Curve | Significance Curve |
| --- | --- | --- |
| Co | y=0.0219e^(-0.089x) | y=0.0219e^(-0.23x) |
| Fe | y=0.0297e^(-0.063x) | y=0.0297e^(-0.1x) |
| Mn | y=0.36e^(-0.072x) | y=0.36e^(-0.09x) |
| Mo | y=0.2408e^(-0.213x) | y=0.2408e^(-0.29x) |
| Ni | y=0.0545e^(-0.074x) | y=0.0545e^(-0.1x) |
| Pb | y=0.1777e^(-0.157x) | y=0.1777e^(-0.21x) |
| U | y=0.1986e^(-0.022x) | y=0.1986e^(-0.03x) |
| V | y=5.6762e^(-0.209x) | y=5.6762e^(-0.28x) |
| W | y=0.2332e^(-0.157x) | y=0.2332e^(-0.2x) |
| Zn | y=0.0808e^(-0.103x) | y=0.0808e^(-0.14x) |

## Slide 2
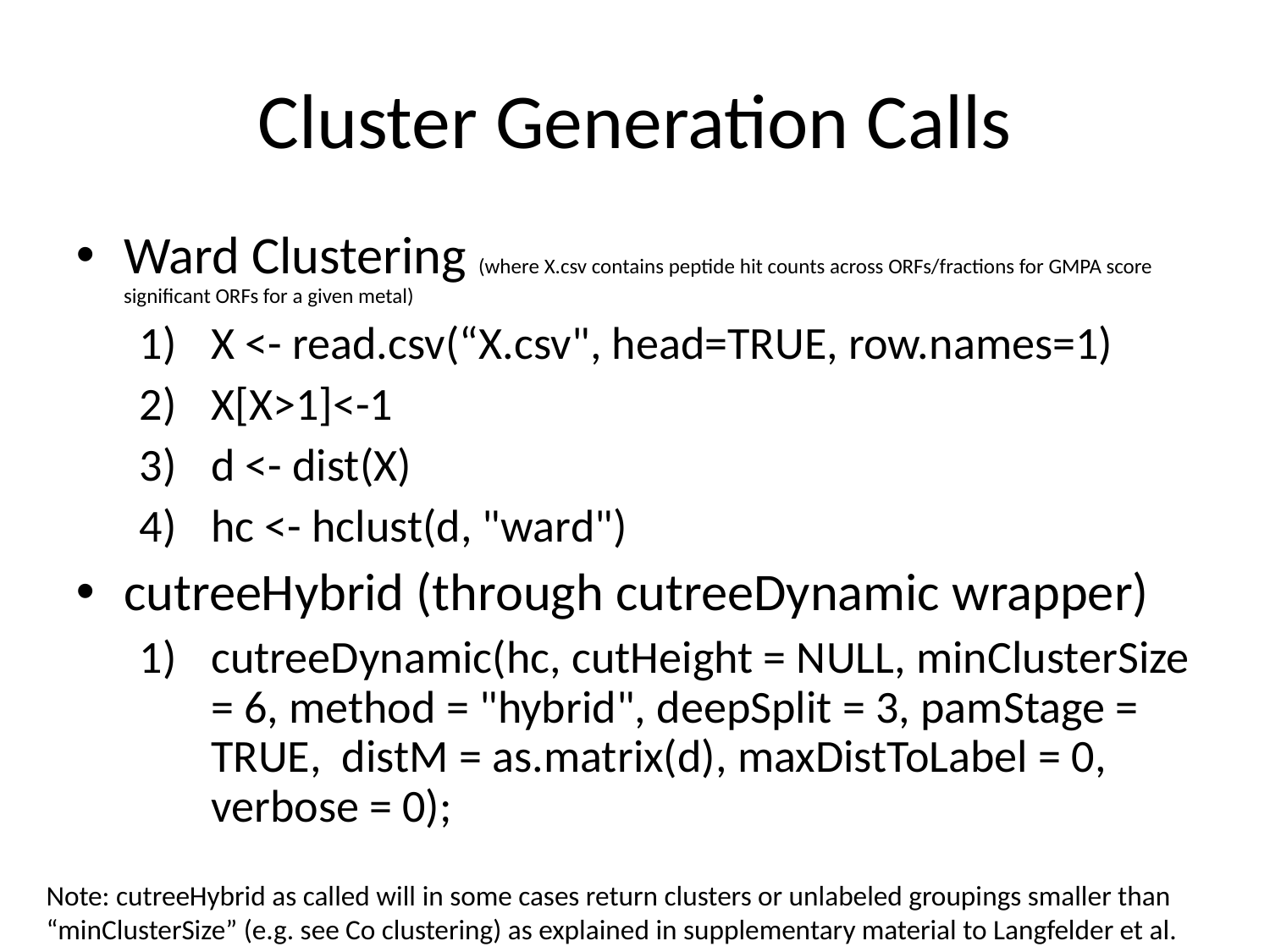

# Cluster Generation Calls
Ward Clustering (where X.csv contains peptide hit counts across ORFs/fractions for GMPA score significant ORFs for a given metal)
X <- read.csv(“X.csv", head=TRUE, row.names=1)
X[X>1]<-1
d <- dist(X)
hc <- hclust(d, "ward")
cutreeHybrid (through cutreeDynamic wrapper)
cutreeDynamic(hc, cutHeight = NULL, minClusterSize = 6, method = "hybrid", deepSplit = 3, pamStage = TRUE, distM = as.matrix(d), maxDistToLabel = 0, verbose = 0);
Note: cutreeHybrid as called will in some cases return clusters or unlabeled groupings smaller than “minClusterSize” (e.g. see Co clustering) as explained in supplementary material to Langfelder et al.
