# Supplementary material for: A Computational Framework for Proteome-Wide Pursuit and Prediction of Metalloproteins using ICP-MS and MS/MS Data
Source: BMC Bioinformatics. 2011 Feb 28;12:64. doi: 10.1186/1471-2105-12-64 (PMC3058030; doi:10.1186/1471-2105-12-64)
Supplement: Additional file 7 — Cluster Diagrams. Tree diagrams of clusters. The colors are used simply to distinguish the defined clusters. [file 1471-2105-12-64-S7.PDF]

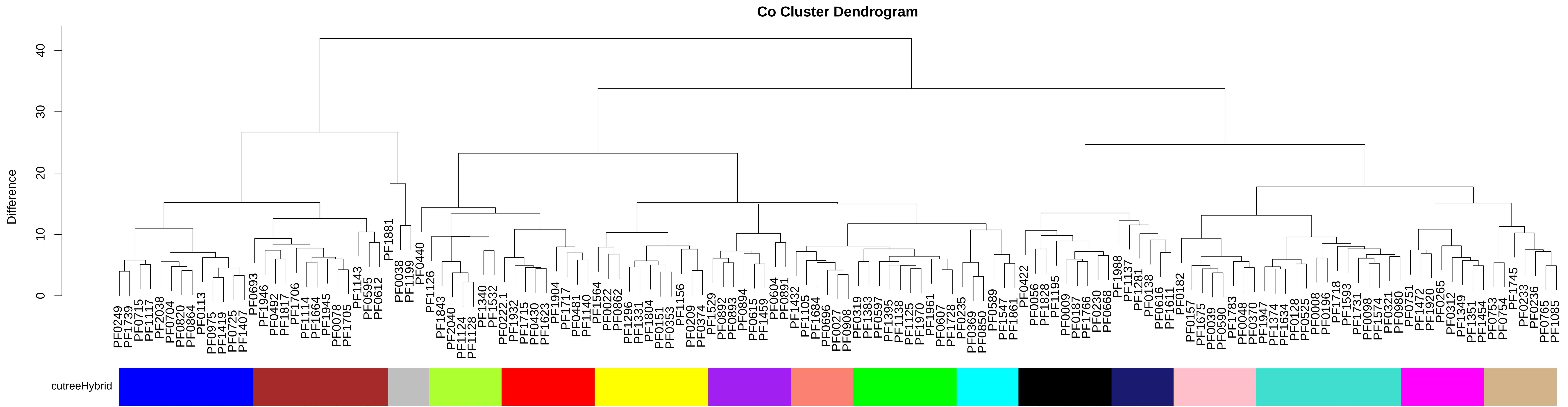
Co Cluster Dendrogram
Difference
0
10
20
30
40
PF0249
PF1739
PF0715
PF1117
PF2038
PF0704
PF0820
PF0864
PF0113
PF0975
PF1419
PF0725
PF1407
PF0693
PF1946
PF0492
PF1817
PF1706
PF1114
PF1664
PF1945
PF0078
PF1705
PF1143
PF0595
PF0612
PF1881
PF0038
PF1199
PF0440
PF1126
PF1843
PF2040
PF1124
PF1128
PF1340
PF1532
PF0222.1
PF1932
PF1715
PF0430
PF1623
PF1904
PF1717
PF0481
PF1140
PF1564
PF0022
PF0862
PF1296
PF1331
PF1804
PF0151
PF0353
PF1156
PF0209
PF0374
PF1529
PF0892
PF0893
PF0894
PF0615
PF1459
PF0604
PF0891
PF1432
PF1105
PF1684
PF0696
PF0027
PF0908
PF0319
PF1383
PF0597
PF1395
PF1138
PF1125
PF1970
PF1961
PF0607
PF1728
PF0235
PF0369
PF0850
PF0589
PF1547
PF1861
PF0422
PF0056
PF1828
PF1195
PF0009
PF0187
PF1766
PF0230
PF0668
PF1988
PF1137
PF1281
PF0138
PF0616
PF1611
PF0182
PF0157
PF1675
PF0039
PF0590
PF1783
PF0048
PF0370
PF1947
PF1374
PF1634
PF0128
PF0525
PF0008
PF0196
PF1718
PF1593
PF1731
PF0098
PF1574
PF0321
PF0980
PF0751
PF1472
PF1920
PF0265
PF0312
PF1349
PF1351
PF1454
PF0753
PF0754
PF1745
PF0233
PF0236
PF0765
PF1085
cutreeHybrid

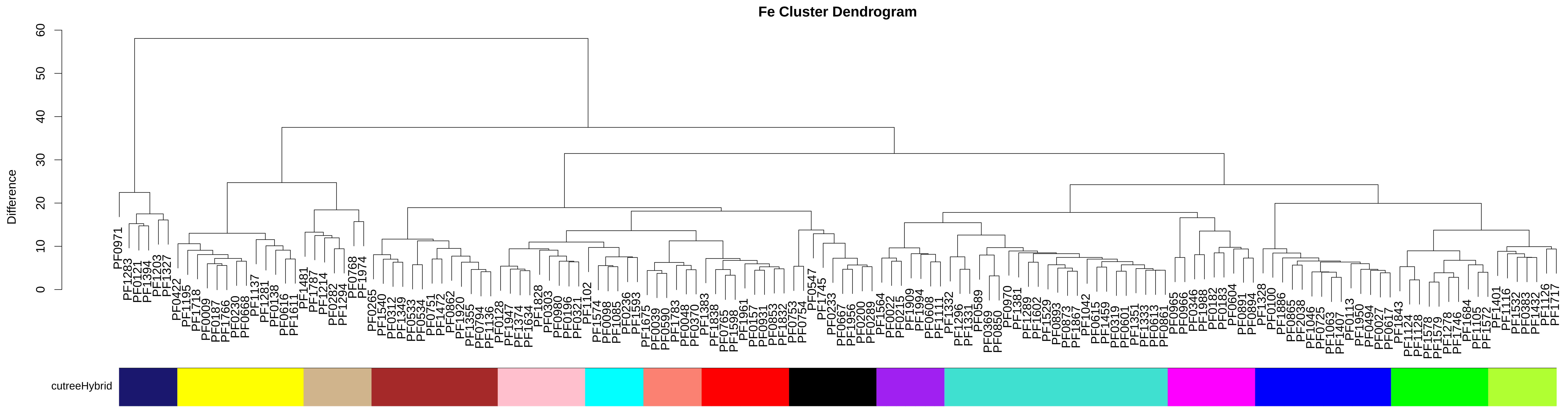
Fe Cluster Dendrogram
Difference
0
10
20
30
40
50
60
cutreeHybrid

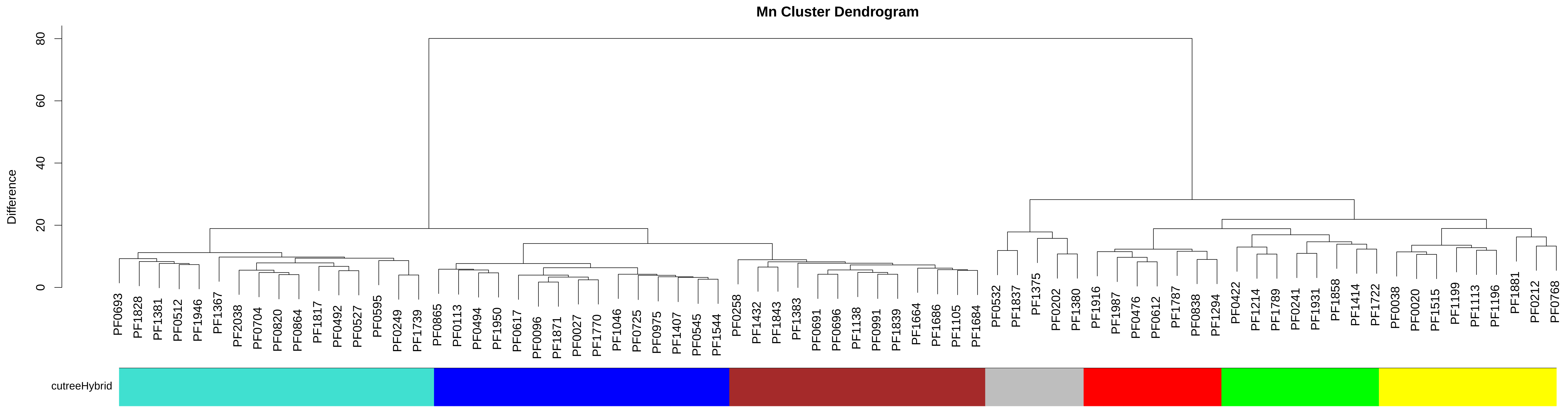
Mn Cluster Dendrogram
Difference
0
20
40
60
80
PF0693
PF1828
PF1381
PF0512
PF1946
PF1367
PF2038
PF0704
PF0820
PF0864
PF1817
PF0492
PF0527
PF0595
PF0249
PF1739
PF0865
PF0113
PF0494
PF1950
PF0617
PF0096
PF1871
PF0027
PF1770
PF1046
PF0725
PF0975
PF1407
PF0545
PF1544
PF0258
PF1432
PF1843
PF1383
PF0691
PF0696
PF1138
PF0991
PF1839
PF1664
PF1686
PF1105
PF1684
PF0532
PF1837
PF1375
PF0202
PF1380
PF1916
PF1987
PF0476
PF0612
PF1787
PF0838
PF1294
PF0422
PF1214
PF1789
PF0241
PF1931
PF1858
PF1414
PF1722
PF0038
PF0020
PF1515
PF1199
PF1113
PF1196
PF1881
PF0212
PF0768
cutreeHybrid

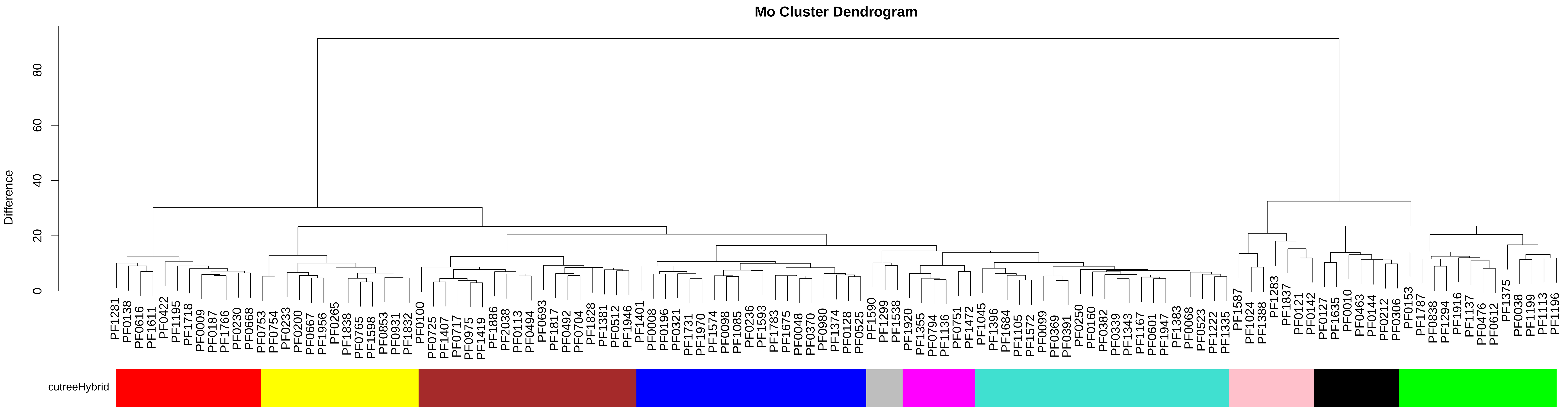

Mo Cluster Dendrogram
Difference
0
20
40
60
80
PF1281 PF0138 PF0616 PF1611 PF0422 PF1195 PF1718 PF0009 PF0187 PF1766 PF0230 PF0668 PF0753 PF0754 PF0233 PF0200 PF0667 PF1956 PF0265 PF1838 PF0765 PF1598 PF0853 PF0931 PF1832 PF0100 PF0725 PF1407 PF0717 PF0975 PF1419 PF1886 PF2038 PF0113 PF0494 PF0693 PF1817 PF0492 PF0704 PF1828 PF1381 PF0512 PF1946 PF1401 PF0008 PF0196 PF0321 PF1731 PF1970 PF1574 PF0098 PF1085 PF0236 PF1593 PF1783 PF1675 PF0048 PF0370 PF0980 PF1374 PF0128 PF0525 PF1590 PF1299 PF1538 PF1920 PF1355 PF0794 PF1136 PF0751 PF1472 PF1045 PF1396 PF1684 PF1105 PF1572 PF0099 PF0369 PF0391 PF0250 PF0160 PF0382 PF0339 PF1343 PF1167 PF0601 PF1947 PF1383 PF0068 PF0523 PF1222 PF1335 PF1587 PF1024 PF1388 PF1283 PF1837 PF0121 PF0142 PF0127 PF1635 PF0010 PF0463 PF0144 PF0212 PF0306 PF0153 PF1787 PF0838 PF1294 PF1916 PF1137 PF0476 PF0612 PF1375 PF0038 PF1199 PF1113 PF1196
cutreeHybrid

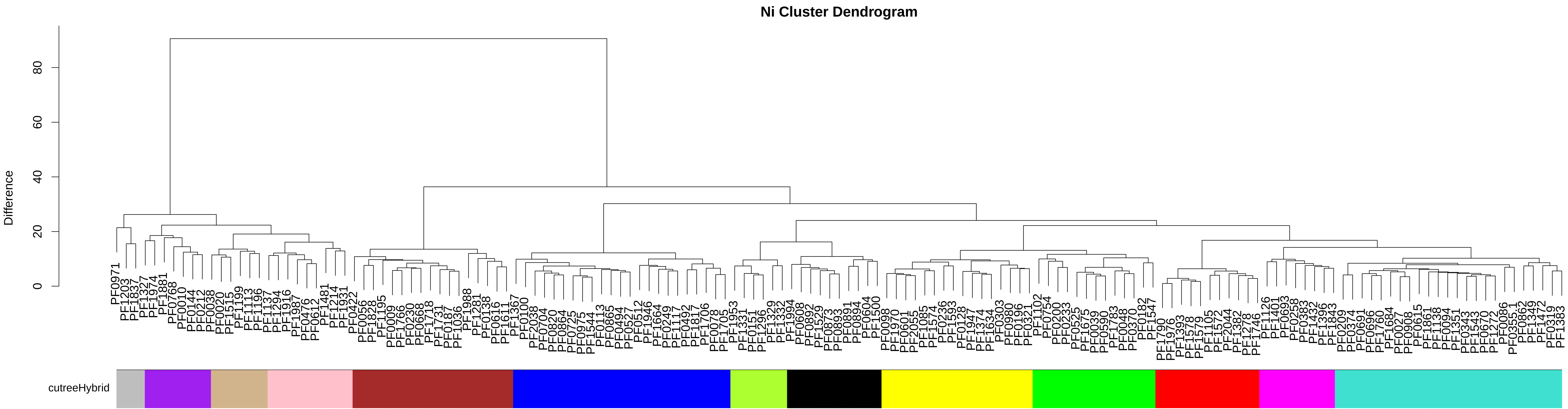

Ni Cluster Dendrogram
Difference
0
20
40
60
80
cutreeHybrid
PF0971 PF1203 PF1837 PF1327 PF1974 PF1881 PF0768 PF0010 PF0144 PF0212 PF0038 PF0020 PF1515 PF1199 PF1113 PF1196 PF1137 PF1294 PF1916 PF1987 PF0476 PF0612 PF1481 PF1214 PF1931 PF0422 PF0056 PF1828 PF1195 PF0009 PF1766 PF0230 PF0668 PF1718 PF1731 PF0187 PF1036 PF1988 PF1281 PF0138 PF0616 PF1611 PF1367 PF0100 PF2038 PF0704 PF0820 PF0864 PF0725 PF0975 PF1544 PF0113 PF0865 PF0494 PF0527 PF0512 PF1946 PF1664 PF0249 PF1117 PF0492 PF1817 PF1706 PF0078 PF1705 PF1953 PF1331 PF0151 PF1296 PF1329 PF1332 PF1994 PF0608 PF0892 PF1529 PF0873 PF0893 PF0881 PF0894 PF0604 PF1500 PF0098 PF1970 PF0601 PF2055 PF1085 PF1574 PF0236 PF1593 PF0128 PF1947 PF1374 PF1634 PF0303 PF0980 PF0196 PF0321 PF1102 PF0754 PF0200 PF0233 PF0525 PF1675 PF0039 PF0590 PF1783 PF0048 PF0370 PF0182 PF1547 PF1790 PF1976 PF1393 PF1578 PF1579 PF1105 PF1572 PF2044 PF1382 PF1278 PF1746 PF1126 PF1401 PF0693 PF0258 PF0383 PF1432 PF1396 PF1843 PF0209 PF0374 PF0691 PF0996 PF1760 PF1684 PF0027 PF0908 PF0615 PF1861 PF1138 PF0094 PF1351 PF0343 PF1543 PF0670 PF1272 PF0086 PF03551 PF0862 PF1349 PF1472 PF0319 PF1383

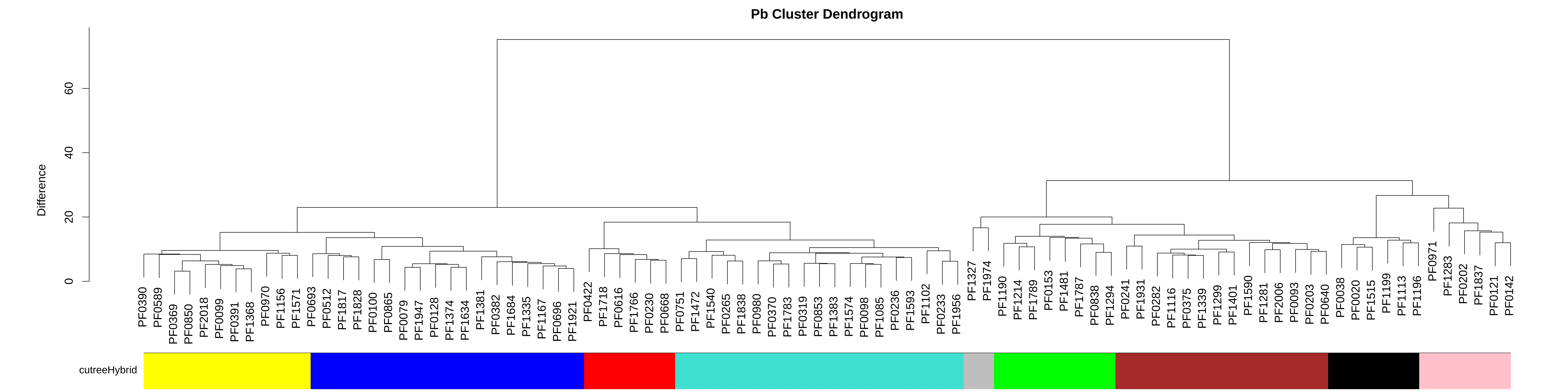
Pb Cluster Dendrogram
Difference
0
20
40
60
PF0390
PF0589
PF0369
PF0850
PF2018
PF0099
PF0391
PF1368
PF0970
PF1156
PF1571
PF0693
PF0512
PF1817
PF1828
PF0100
PF0865
PF0079
PF1947
PF0128
PF1374
PF1634
PF1381
PF0382
PF1684
PF1335
PF1167
PF0696
PF1921
PF0422
PF1718
PF0616
PF1766
PF0230
PF0668
PF0751
PF1472
PF1540
PF0265
PF1838
PF0980
PF0370
PF1783
PF0319
PF0853
PF1383
PF1574
PF0098
PF1085
PF0236
PF1593
PF1102
PF0233
PF1956
PF1327
PF1974
PF1190
PF1214
PF1789
PF0153
PF1481
PF1787
PF0838
PF1294
PF0241
PF1931
PF0282
PF1116
PF0375
PF1339
PF1299
PF1401
PF1590
PF1281
PF2006
PF0093
PF0203
PF0640
PF0038
PF0020
PF1515
PF1199
PF1113
PF1196
PF0971
PF1283
PF0202
PF1837
PF0121
PF0142
cutreeHybrid

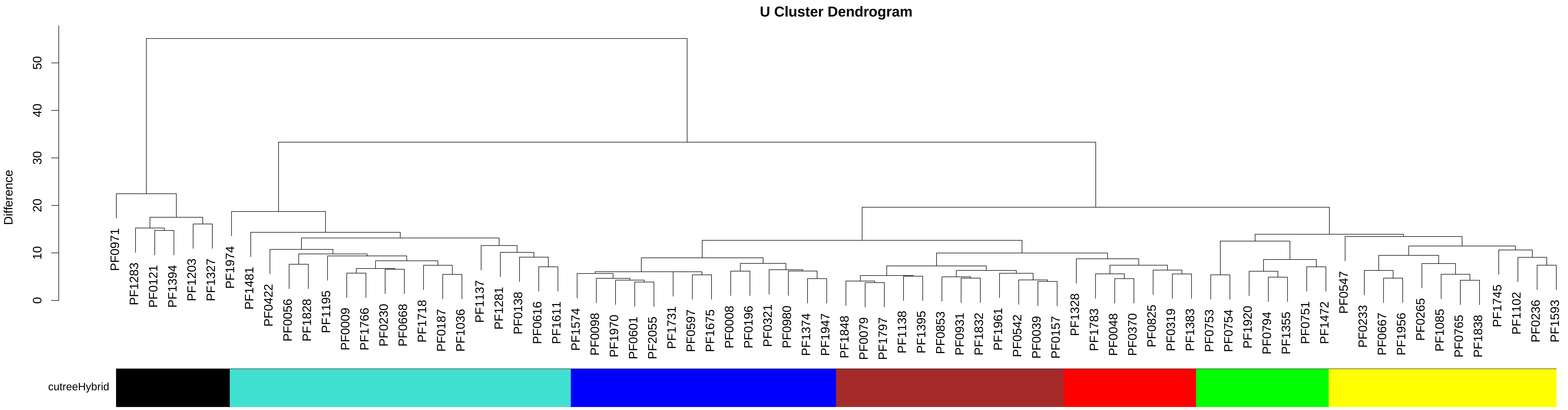
U Cluster Dendrogram
Difference
0
10
20
30
40
50
PF0971
PF1283
PF0121
PF1394
PF1203
PF1327
PF1974
PF1481
PF0422
PF0056
PF1828
PF1195
PF0009
PF1766
PF0230
PF0668
PF1718
PF0187
PF1036
PF1137
PF1281
PF0138
PF0616
PF1611
PF1574
PF0098
PF1970
PF0601
PF2055
PF1731
PF0597
PF1675
PF0008
PF0196
PF0321
PF0980
PF1374
PF1947
PF1848
PF0079
PF1797
PF1138
PF1395
PF0853
PF0931
PF1832
PF1961
PF0542
PF0039
PF0157
PF1328
PF1783
PF0048
PF0370
PF0825
PF0319
PF1383
PF0753
PF0754
PF1920
PF0794
PF1355
PF0751
PF1472
PF0547
PF0233
PF0667
PF1956
PF0265
PF1085
PF0765
PF1838
PF1745
PF1102
PF0236
PF1593
cutreeHybrid

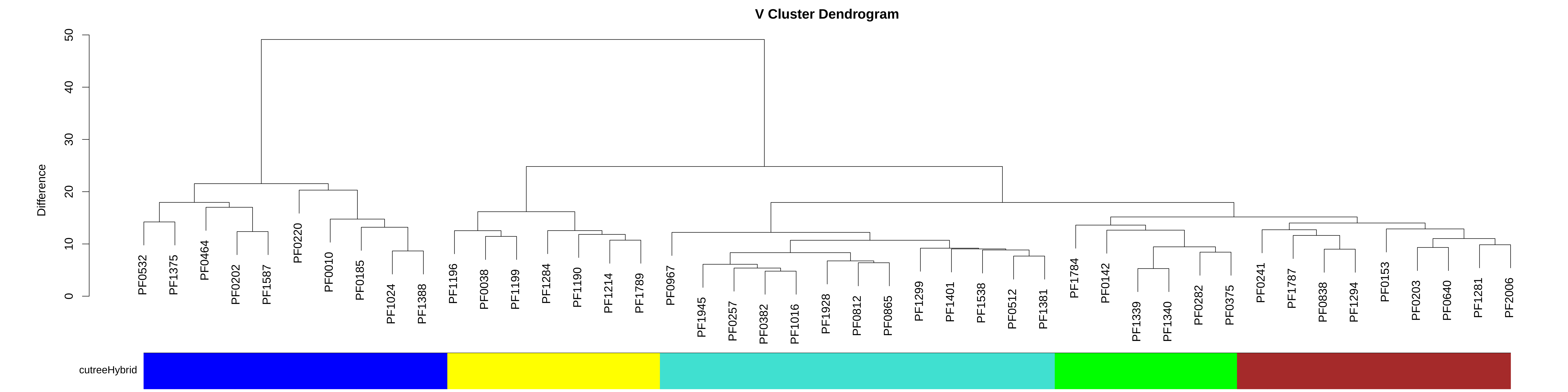
V Cluster Dendrogram
Difference
0
10
20
30
40
50
PF0532
PF1375
PF0464
PF0202
PF1587
PF0220
PF0010
PF0185
PF1024
PF1388
PF1196
PF0038
PF1199
PF1284
PF1190
PF1214
PF1789
PF0967
PF1945
PF0257
PF0382
PF1016
PF1928
PF0812
PF0865
PF1299
PF1401
PF1538
PF0512
PF1381
PF1784
PF0142
PF1339
PF1340
PF0282
PF0375
PF0241
PF1787
PF0838
PF1294
PF0153
PF0203
PF0640
PF1281
PF2006
cutreeHybrid

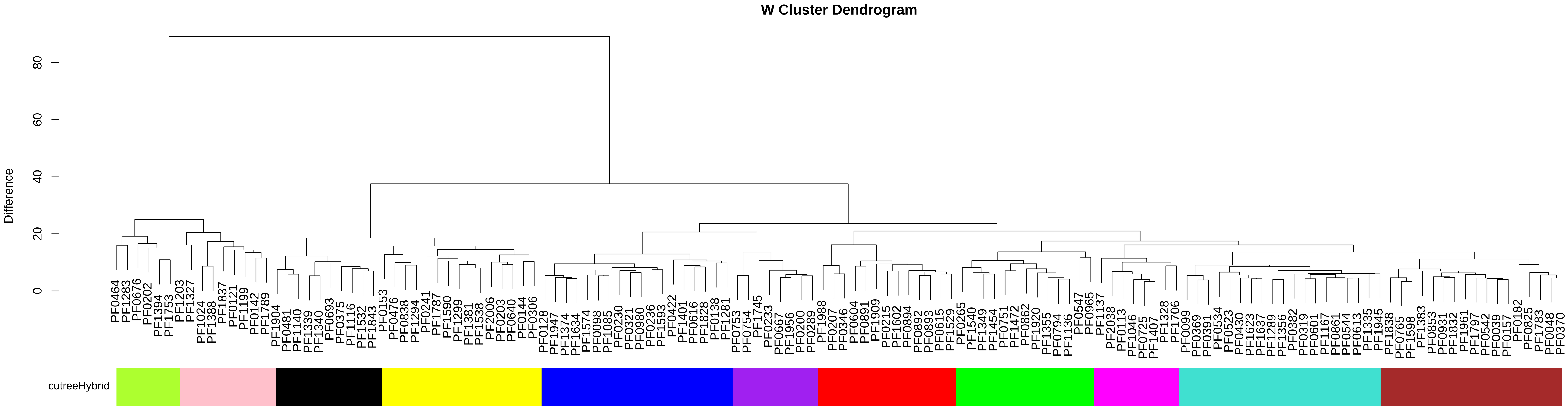
W Cluster Dendrogram
Difference
0
20
40
60
80
PF0464 PF1283 PF0676 PF0202 PF1394 PF1753 PF1203 PF1327 PF1024 PF1388 PF1837 PF0121 PF1199 PF0142 PF1789 PF1904 PF0481 PF1140 PF1339 PF1340 PF0693 PF0375 PF1116 PF1532 PF1843 PF0153 PF0476 PF0838 PF1294 PF0241 PF1787 PF1590 PF1299 PF1381 PF1538 PF2006 PF0203 PF0640 PF0144 PF0306 PF0128 PF1947 PF1374 PF1634 PF1574 PF0098 PF1085 PF0230 PF0321 PF0980 PF0236 PF1593 PF0422 PF1401 PF0616 PF1828 PF0138 PF1281 PF0753 PF0754 PF1745 PF0233 PF0667 PF1956 PF0200 PF0289 PF1988 PF0207 PF0346 PF0604 PF0891 PF1909 PF0215 PF1602 PF0894 PF0892 PF0893 PF0615 PF1529 PF0265 PF1540 PF1349 PF1454 PF0751 PF1472 PF0862 PF1920 PF1355 PF0794 PF1136 PF0547 PF0965 PF1137 PF2038 PF0113 PF1046 PF0725 PF1407 PF1328 PF1706 PF0099 PF0369 PF0391 PF0534 PF0523 PF0430 PF1623 PF1637 PF1289 PF1356 PF0382 PF0319 PF0601 PF1167 PF0861 PF0544 PF0613 PF1335 PF1945 PF1838 PF0765 PF1598 PF1383 PF0853 PF0931 PF1832 PF1961 PF1797 PF0542 PF0039 PF0157 PF0182 PF0825 PF1783 PF0048 PF0370
cutreeHybrid

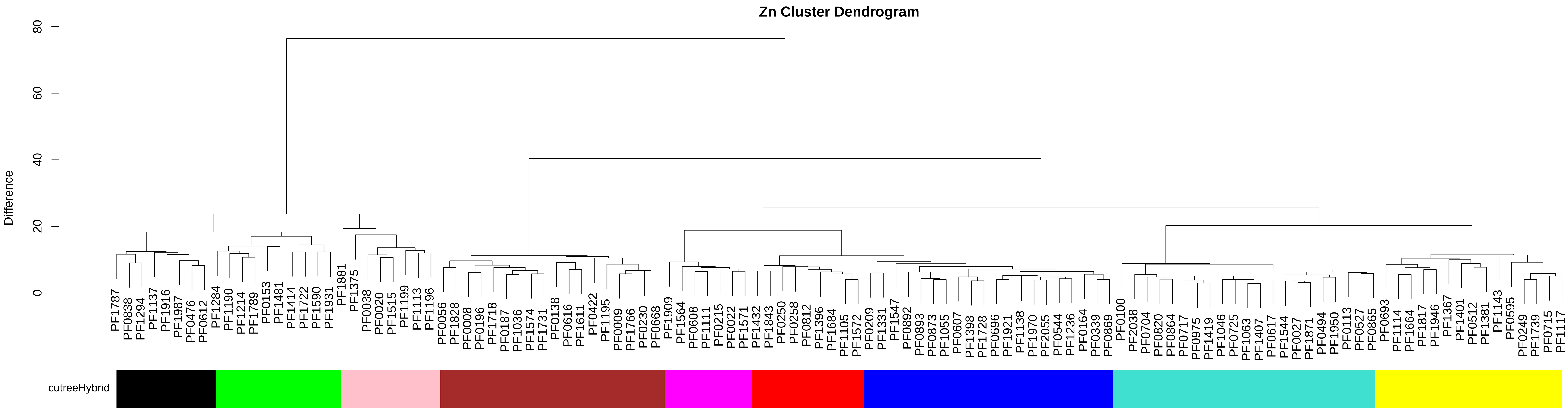
Zn Cluster Dendrogram
Difference
0
20
40
60
80
PF1787
PF0838
PF1294
PF1137
PF1916
PF1987
PF0476
PF0612
PF1284
PF1190
PF1214
PF1789
PF0153
PF1481
PF1414
PF1722
PF1590
PF1931
PF1881
PF1375
PF0038
PF0020
PF1515
PF1199
PF1113
PF1196
PF0056
PF1828
PF0008
PF0196
PF1718
PF0187
PF1036
PF1574
PF1731
PF0138
PF0616
PF1611
PF0422
PF1195
PF0009
PF1766
PF0230
PF0668
PF1909
PF1564
PF0608
PF1111
PF0215
PF0022
PF1571
PF1432
PF1843
PF0250
PF0258
PF0812
PF1396
PF1684
PF1105
PF1572
PF0209
PF1331
PF1547
PF0892
PF0893
PF0873
PF1055
PF0607
PF1398
PF1728
PF0696
PF1921
PF1138
PF1970
PF2055
PF0544
PF1236
PF0164
PF0339
PF0869
PF0100
PF2038
PF0704
PF0820
PF0864
PF0717
PF0975
PF1419
PF1046
PF0725
PF1063
PF1407
PF0617
PF1544
PF0027
PF1871
PF0494
PF1950
PF0113
PF0527
PF0865
PF0693
PF1114
PF1664
PF1817
PF1946
PF1367
PF1401
PF0512
PF1381
PF1143
PF0595
PF0249
PF1739
PF0715
PF1117
cutreeHybrid
